# Engineering Cardiac Small Extracellular Vesicle-Derived Vehicles with Thin-Film Hydration for Customized microRNA Loading

**DOI:** 10.3390/jcdd8110135

**Published:** 2021-10-22

**Authors:** Sruti Bheri, Brandon P. Kassouf, Hyun-Ji Park, Jessica R. Hoffman, Michael E. Davis

**Affiliations:** 1 Wallace. H. Coulter Department of Biomedical Engineering, Georgia Institute of Technology & Emory University School of Medicine, Atlanta, GA 30332, USA; srutibheri@gatech.edu (S.B.); kassoufbrandon@gatech.edu (B.P.K.); hyunji.park@emory.edu (H.-J.P.); jrhoff3@emory.edu (J.R.H.); 2 Children’s Heart Research and Outcomes (HeRO) Center, Children’s Healthcare of Atlanta & Emory University, Atlanta, GA 30322, USA

**Keywords:** extracellular vesicle, vesicle engineering, exosome, cardiac ckit+ progenitor cell, cardiac repair, thin-film hydration, microRNA-126

## Abstract

Cell therapies for myocardial infarction, including cardiac ckit+ progenitor cell (CPC) therapies, have been promising, with clinical trials underway. Recently, paracrine signaling, specifically through small extracellular vesicle (sEV) release, was implicated in cell-based cardiac repair. sEVs carry cardioprotective cargo, including microRNA (miRNA), within a complex membrane and improve cardiac outcomes similar to that of their parent cells. However, miRNA loading efficiency is low, and sEV yield and cargo composition vary with parent cell conditions, minimizing sEV potency. Synthetic mimics allow for cargo-loading control but consist of much simpler membranes, often suffering from high immunogenicity and poor stability. Here, we aim to combine the benefits of sEVs and synthetic mimics to develop sEV-like vesicles (ELVs) with customized cargo loading. We developed a modified thin-film hydration (TFH) mechanism to engineer ELVs from CPC-derived sEVs with pro-angiogenic miR-126 encapsulated. Characterization shows miR-126+ ELVs are similar in size and structure to sEVs. Upon administration to cardiac endothelial cells (CECs), ELV uptake is similar to sEVs too. Further, when functionally validated with a CEC tube formation assay, ELVs significantly improve tube formation parameters compared to sEVs. This study shows TFH-ELVs synthesized from sEVs allow for select miRNA loading and can improve in vitro cardiac outcomes.

## 1. Introduction

Myocardial infarction (MI) is one of the leading causes of morbidity and mortality worldwide with an estimated 0.8 million events occurring annually in the United States alone [1]. MI involves the onset of cardiac ischemia following coronary artery occlusion. At a cellular level, ischemia leads to hypoxia which initiates an inflammatory response and activates neovascularization and fibroblast-activated scar formation [2,3,4]. Despite these attempts at local tissue repair, ischemia leads to irreversible myocardial damage, unfavorable cardiac remodeling and eventually results in end-stage cardiac failure. To enable cardiac recovery after MI, rapid medical intervention is crucial. Treatments include prompt reperfusion and revascularization through pharmacological agents, antithrombotic therapies, or mechanical interventions such as angioplasty and stenting [5]. Beyond these traditional approaches, cell-based therapies have shown promise, with the scope to induce cardiac repair and recovery when delivered to the injured site [6,7]. Specifically, cardiac-derived ckit+ progenitor cells (CPCs) were implicated as pro-reparative agents for cardiac recovery with phase II clinical trials completed (NCT02501811) [8,9,10].

More recently, the benefits of cell-based myocardial therapies were attributed to paracrine signaling, specifically through small extracellular vesicle (sEV) release [11,12]. sEVs are 30–180 nm vesicles carrying nucleic acid cargo, such as RNA and microRNA (miRNA), enveloped in an amphiphilic lipid and protein bilayer [13]. Their cargo is often enriched in specific RNA compared to the parent cell and sEVs can be similar to or even more effective than parent cells in inducing cardiac repair [14,15]. However, current sEV therapies depend on parent cell-based sEV release, which can vary with the cellular microenvironment and the state of the cell [16,17]. Consequently, there is variability in sEV yield, physiochemical properties, and loaded cargo [18]. Moreover, specific parameters such as CPC-donor age, CPC aggregation, and hypoxia impact sEV release, alter the cargo, and in turn affect cardiac function [17,19,20]. The CPC-based sEV therapies also depend on in vivo cell survival, retention, and functionality. Finally, even if sEVs are successfully delivered and retained in the injured myocardium, the fraction of total sEV cargo that is cardio-protective is low, thereby minimizing the potency sEVs [21]. Therefore, despite the observed benefits of sEVs for cardiac repair, there remains a need for reliable sEV-based therapies with optimized, high concentration cargo to ensure potent and lasting reparative effects after MI.

To address the parent cell dependency and cargo variabilities of sEV therapeutics, synthetic mimics were designed. These can be grossly divided into exogenously modified sEVs and synthetic nanoparticles such as small unilamellar vesicles (SUVs). Exogenously modified sEVs focus on modifying the cargo by adding therapeutic agents into the vesicle via active or passive encapsulation [22,23,24]. Although some functional molecules were delivered using these methods, they have high immunogenicity and compromised vesicle membranes [25,26]. SUVs on the sEV-scale were more promising, composed of either synthetic or chemically derived natural lipids. These vesicles are biocompatible, have the potential to carry tailored cargo and can be engineered for cell-specific targeting [27]. However, SUV membranes usually consist of only a couple of lipid types, unlike sEVs which have complex, multi-molecule-based membranes [28,29]. This often leads to SUVs with low solubility and stability, and rapid clearance from tissues [29,30,31]. Thus, despite their cargo customizability, SUVs fall short of matching the in vivo cardiac reparative potential of sEVs.

Here, we aimed to engineer an sEV-like vesicle (ELV) by combining the benefits of sEV membranes with the cargo loading capacity of synthetic mimics. Previous methods to exogenously modify vesicles have included sonication, extrusion, electroporation, freeze–thaw cycling, and forming cell-membrane-derived vesicles [32]. However, these processes have shown variable successes with cargo loading and can induce cytotoxicity [22,25]. Further, some methods involving a temporary rupture and resealing of the membrane can compromise membrane integrity and increase vesicle aggregation [33]. An alternative that is well established for synthetic nanoparticle design from artificial lipids is thin-film hydration (TFH) [34,35,36]. This method involves the formation of a lipid film followed by rehydration with an aqueous solvent to form vesicles. Although this is primarily limited to synthetic nanoparticles, this allows for higher cargo loading efficiency and even allows for membrane lipid modifications unlike most exogenously modified sEV methods [37]. Given the versatility and cargo loading ability of TFH, we chose this approach for designing our ELVs.

The objective of this work was to develop scalable and potent vesicle therapies for cardiac repair, specifically through the loading of miRNA. More specifically, we aimed to (1) synthesize ELVs from CPC-sEV membranes using a modified TFH method and (2) allow for customized miRNA cargo loading into ELVs. Our findings show that CPC-sEV derived ELVs can successfully be formed on the sEV scale with miR-126 cargo encapsulated. Further, we show ELVs can be internalized and have pro-angiogenic potential compared to sEVs when administered to cardiac endothelial cells (CECs). This study provides the groundwork for developing ELV-based therapies for highly potent and tunable RNA delivery after the onset of MI.

## 2. Materials and Methods

### 2.1. Isolation and Culture of Human CPCs

Human CPCs were isolated from the right atrial appendage tissue of neonatal pediatric patients, through CD-177 magnetic bead sorting as described previously [38]. Neonate patients were classified as patients <1 week old at the time of appendage removal during surgical intervention for a congenital heart defect. CPCs were cultured in Ham’s F-12 medium (Corning Cellgro^®^, Corning, NY, USA) with 10% fetal bovine serum (FBS), 1% penicillin-streptomycin, 1% L-glutamine and 0.04% human fibroblast growth factor-β (hFGF-β).

### 2.2. Culture of Rat CECs

Rat CECs were cultured in endothelial growth medium (EGM-2 Endothelial Cell Growth Medium-2 BulletKit^TM^, Lonza, Bend, OR) supplemented with 1% penicillin-streptomyocin and 2% FBS, 0.4% hFGF-β, 0.1% vascular endothelial growth factor (VEGF), 0.1% long arginine 3 insulin-like growth factor (R3-IGF-1), 0.1% ascorbic acid, 0.1% human epidermal growth factor (hEGF), 0.1% Gentamicin/Amphotericin-B (GA-1000), 0.1% heparin, and 0.04% hydrocortisone as per manufacturer’s protocol.

### 2.3. Isolation and Characterization of sEVs

2D cultures of CPCs (~100 × 10^6^ cells) between passages 9–13 were grown to 90% confluency. When confluent, CPCs were transferred to FBS-depleted media and their conditioned media was collected after 24 h. sEVs were isolated from conditioned media using differential ultracentrifugation (Optima XPN-100, Beckman Coulter, Indianapolis, IN, USA). The CPC conditioned media was sequentially depleted of cells at 1000 RPM for 5 min (Centrifuge 5810 R, Eppendorf, Hamburg, Germany) and cell debris at 31,000 RPM for 20 min (SW32Ti, Beckman Coulter) after which sEVs were pelleted at 31,000 RPM for 114 min (SW41Ti, Beckman Coulter) [39]. sEV pellets were collected and resuspended in PBS as required and stored at −80 °C until further use. sEV shape was assessed with transmission electron microscopy (JEOL JEM-1400, Peabody, MA, USA), particle size and concentration determined through Nanoparticle Tracking Analysis (NanoSight NS-300 with NTA 3.4 software, Malvern Panalytical, Malvern, UK) and polydispersity index through Dynamic Light Scattering (DynaPro Plate Reader III, Wyatt, Santa Barbara, CA, USA).

### 2.4. Formation of sEV Lipid Bilayer

ELVs were synthesized from sEVs using a variation of the TFH method, a commonly used process for synthetic vesicle formation. Specifically, the TFH method was modified to allow lipid film creation directly from sEVs instead of from synthetic lipids, as is required for traditional TFH. For this, inherent sEV cargo was removed using repeated sonication and flash freeze–thaw cycling. Samples were sonicated at #3 with probe sonicator (Sonic Dismembrator Model 100, Thermo Fisher Scientific, Waltham, MA, USA) for 30 s (in sets of 10 s) followed by rapid freezing in liquid nitrogen. Samples were then rapidly reheated in a water bath (80 °C), and this sonication-freeze–thaw cycle was repeated 5 times. Samples were then transferred to 10 mL single neck round bottom flasks (14/20 joint, Corning) along with 1 mL chloroform to confirm initial sample evaporation during TFH. The rotary evaporator (Rotovapor R-100, BUCHI, New Castle, DE, USA) was set up with the heating bath at 50 °C (Heating Bath B-100, BUCHI) and fresh dry ice and acetone in the condenser. The flask was secured to rotary evaporator and initial chloroform was evaporated at 332 mbar vacuum followed by aqueous sEV buffer evaporation at 42 mbar vacuum. Sample was submerged into the heating bath for complete aqueous solvent evaporation, as required, and left to dry for 10–15 min. Once thoroughly dried, 1–2 mL chloroform was again added to flask and left to rotate at #5 for 30 min to allow the sEV membrane to dissolve. After 30 min, rotary evaporation was repeated until all chloroform was evaporated, and a uniform lipid film formed in the flask. Samples were then desiccated overnight at room temperature to remove any trace solvents.

### 2.5. Synthesis of miR-loaded ELVs

Desiccated sEV samples (from 2.4.) were treated with 1 µg/mL RNase A (Thermo Fisher Scientific) and incubated with rotation at room temperature for 30 min to deplete inherent sEV RNA cargo. Sample was then dried by rotary evaporation in 50 °C water bath and 42 mbar vacuum to remove aqueous buffer. A total of 1 mL chloroform was again added and incubated at room temperature for 30 min followed by evaporation (332 mbar) to dissolve lipids and reform a lipid film. Samples were then incubated with 40 units/20 µL ribonuclease inhibitor (RNaseOUT, Invitrogen, Carlsbad, CA, USA) and 1 mM DTT (Invitrogen) for 1 h at 37 °C followed by evaporation to inhibit further RNase A activity. Again, 1 mL chloroform was added and evaporated after a 10-min incubation to reform lipid film. Samples were run through inert gas and desiccated overnight to stabilize and remove trace chemicals. Desiccated samples were loaded with miR-126-5p at 200 µg/mL, vortexed for 10 s and incubated at 4°C overnight, with shaking, to allow lipid film to rehydrate and ELVs to self-assemble in aqueous medium [21]. Following this, PBS was added to samples to dilute excess glycerol from RNaseOUT to ≤3% and provide ELV stability. Samples were passed under inert gas and centrifuged at 1000 RPM for 5 min to deplete larger particles (Centrifuge 5810 R, Eppendorf). To remove unencapsulated miRNA and concentrate the samples, samples were ultracentrifuged at 31,000 RPM for 114 min (SW41Ti, Beckman Coulter) and resuspended in PBS as needed.

### 2.6. RNA Isolation and ELV Cargo Quantification

RNA was isolated from 1.00 × 10^6^ particles of ELVs or sEVs using the miRNeasy Micro Kit (Qiagen, Germantown, MD, USA) as per manufacturer’s protocol. Total isolated RNA concentration was quantified (NanoDrop One, Thermo Fisher Scientific) and miR-126 encapsulation in ELV and sEV groups was detected through standard curve Real-Time quantitative polymerase chain reaction (RT-qPCR) on a StepOnePlus system (Applied Biosystems, Foster City, CA, USA). Data were represented as miR-126 RNA mass.

### 2.7. ELV Internalization

ELV internalization by CECs was observed by confocal laser scanning microscopy and quantified through flow cytometry. Briefly, CECs cultured until 90% confluency were seeded at 0.20 × 10^6^ cells/mL in 40 µL/well onto 6-channel cell culture slides (IBIDI sticky Slide VI 0.4, Fitchburg, WI, USA) pre-coated with 0.1% gelatin. Slides were incubated for 3–4 h until CECs adhered after which channels were filled until 180 µL to avoid channel drying and incubated at 37 °C overnight. CECs were then quiesced in endothelial bare media (FBS and growth factor free) with 1% penicillin-streptomycin for 12 h. After 12 h, CECs were treated with sEVs or miR-126+ ELVs stained with membrane dye calcein (Thermo Fisher Scientific) at 5.00 × 10^8^ particles per 1.00 × 10^6^ cells and incubated at 37 °C for 2–3 h. Next CECs in channels were fixed with 4% paraformaldehyde and stored at 4 °C. Prior to imaging, CEC were stained with LysoBrite NIR (AAT Bioquest, Sunnyvale, CA, USA) and DAPI (Thermo Fisher Scientific) and incubated at 37 °C for 30 min to visualize vesicle trafficking to the lysosome. Slides were imaged with confocal laser scanning microscope (Olympus FV1000, Center Valley, PA, USA).

For quantification, CECs were cultured until 90% confluency and then seeded at 0.05 × 10^6^ cells/well into 24 well plates pre-coated with 0.1% gelatin. After incubation for attachment, CECs were then quiesced overnight in endothelial bare media (FBS and growth factor free) with 1% penicillin-streptomycin. CECs were treated with calcein-stained sEVs or miR-126+ ELVs at 5.00 × 10^8^ particles per 1.00 × 10^6^ cells and incubated at 37 °C for 2–3 h. CECs were then washed to remove free vesicles, collected, and resuspended in flow buffer (2% FBS in PBS). Internalization of sEVs and ELVs was quantified through flow cytometry (Cytek Aurora, Fremont, CA, USA) for λ_Ex_/λ_Em_ = 495/515 nm. Negative control was CECs treated with calcein-stained ELVs or sEVs and incubated at 4 °C to inhibit uptake.

### 2.8. Tube Formation Assay

CECs were cultured until 90% confluency. CECs were then quiesced in endothelial bare media (FBS and growth factor free) with 1% penicillin-streptomycin. Quiesced CECs were seeded at 10,000 cells/well onto µ-slide Angiogenesis slide (IBIDI) pre-coated with 10 µL/well Matrigel (Matrigel^®^ Matrix, Corning) as per manufacturer’s protocol. CECs were then treated overnight at 5.00 × 10^8^ sEVs or miR-126+ ELVs per 1.00 × 10^6^ cells. CECs were then stained with calcein-AM (Thermo Fisher Scientific) and imaged using a fluorescent microscope (Olympus IX71) such that each image captured one complete well, representing one technical replicate of a sample group. ImageJ software was used to quantify different tube length parameters (Fiji, National Institutes of Health, Bethesda, MD, USA) [40]. The Angiogenesis Analyzer plug-in for ImageJ, specifically created to analyze the vascular organization of endothelial cells, was used to quantify images (three technical replicates per group) [41]. Output parameters of number of tubules, total tube length and total segment length were calculated, with lengths measured in pixels. Negative and positive controls consisted of quiesced CECs with no treatment, and EGM-grown CECs with no treatment, respectively.

### 2.9. Statistical Analysis

All statistical analysis was performed using GraphPad PRISM 8 software (GraphPad, San Diego, CA, USA) with specific testing details outlined in figure captions.

## 3. Results

### 3.1. sEVs Successfully Isolated and Characterized from 2D CPC Cultures

**Figure 1:**
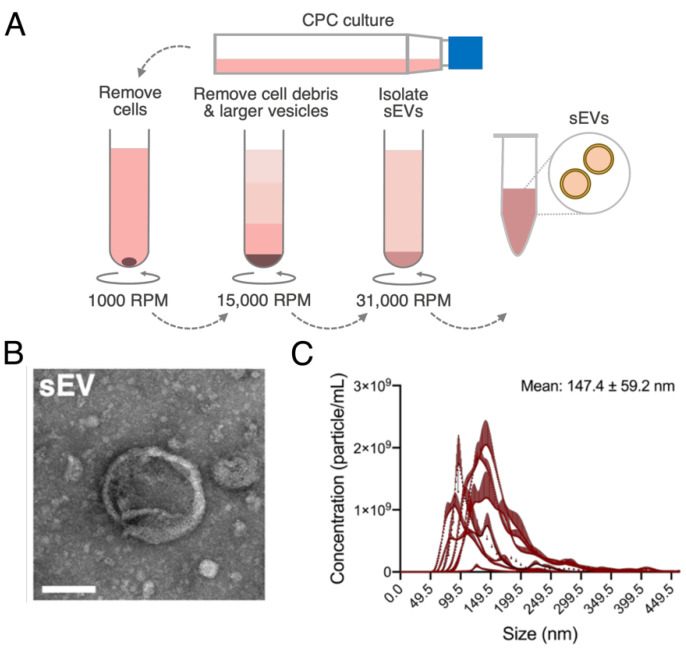
Cardiac ckit+ progenitor cell (CPC)-derived small extracellular vesicle (sEV) isolation and characterization. (**A**) Workflow of sEV isolation from 2D cultures of CPCs with differential ultracentrifugation to sequentially remove cells, cell debris and larger vesicles. (**B**) Transmission electron microscopy image of isolated CPC-sEV. Scale bar = 100 nm. (**C**) Concentration-size profile of isolated sEVs with nanoparticle tracking analysis (NTA). *n* = 11.

Human CPCs were grown in 2D cultures until 90% confluency and sEVs were isolated from CPC conditioned media through differential ultracentrifugation (Figure 1A). sEV shape and presence of the bilayer membrane were detected through transmission electron microscopy (Figure 1B). Further, sEV size was within the expected range (147.4 ± 59.2 nm) and sEVs were isolated at approximately 8.87 × 10^10^ ± 8.42 × 10^9^ particles/mL (Figure 1C).

### 3.2. ELVs on A sEV Scale Successfully Synthesized with TFH with Selective miR Loading

**Figure 2:**
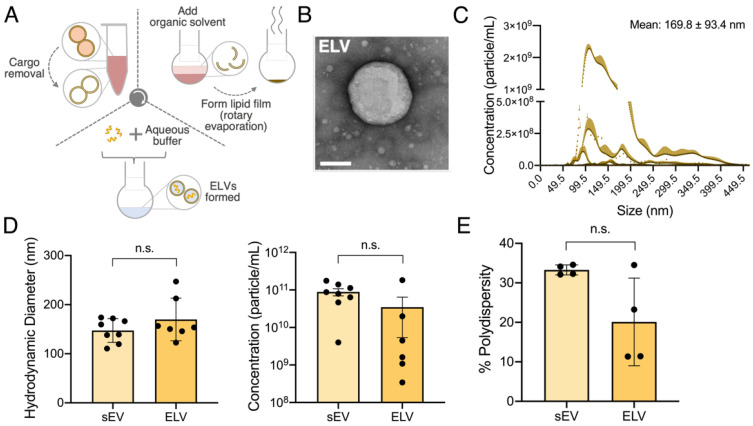
Synthesis and characterization of sEV-derived sEV-like vesicles (ELVs) using a modified thin-film-hydration (TFH) method. (**A**) Workflow of ELV synthesis from CPC-sEVs by depleting sEV cargo, forming a lipid film and rehydrating with customized microRNA (miRNA). (**B**) Transmission-electron microscopy image of an engineered ELV. Scale bar = 100 nm. (**C**) Concentration-size profile of an ELV with NTA. *n* = 10. (**D**) Comparison of ELV and sEV size and concentration. (**E**) Comparison of percentage poly-dispersity of ELVs and sEVs. *n* = 4–8 represent biological replicates. Mean ± SEM. Significance was tested with two-way, Student’s paired *t*-test. n.s. = not significant.

A modified version of the TFH method, used for synthetic vesicle formation, was chosen to synthesize ELVs from sEVs (Figure 2A). Briefly, initial sEV cargo removal was attempted through a combination of sonication and repeated freeze–thaw cycling. Following this, rotary evaporation was used to form a uniform lipid layer to which chosen miRNA, suspended in an aqueous buffer, was added to initiate the self-assembly of ELVs. ELV shape, similar to that of sEVs, was detected through transmission electron microscopy to show the successful formation of vesicles (Figure 2B). The size profiles of ELVs were similar to that of sEVs (169.8 ± 93.4 nm vs. 147.4 ± 59.2 nm for sEVs) with no significant difference observed (Figure 2C,D). Further, ELV concentration was similar to that of sEVs at 3.49 × 10^10^ ± 3.14 × 10^9^ particles/mL, although there was greater batch-to-batch variation observed (Figure 2C,D). This variability is likely due to sampling loss during the multi-step ELV synthesis. The polydispersity index of ELVs was 0.20 ± 0.055, suggesting a uni-modal population, and that of sEVs was 0.33 ± 0.006 with no statistical difference between the two groups (Figure 2E). Overall, ELV external structure matched that of the sEVs.

**Figure 3:**
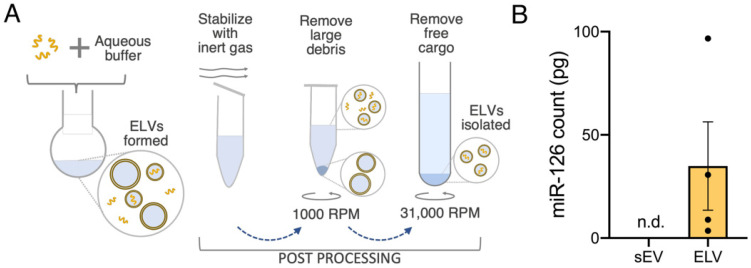
miR-126 was encapsulated into ELVs with the modified TFH. (**A**) Workflow of ELV post-processing to stabilize ELVs, remove larger debris and cargo-free vesicles, remove free unencapsulated miRNA cargo and isolate miRNA+ELVs. (**B**) Quantification of endothelial-specific miR-126 loading into ELVs as compared to presence in sEVs as counts per 1.00 × 10^6^ particles. *n* = 4 represent biological replicates. Mean ± SEM. n.d. = not detected.

To demonstrate cargo loading ability, miR-126-5p was loaded into the ELVs. miR-126 was specifically chosen as it has low abundance in CPC-sEVs but is implicated in cardioprotective endothelial function, so its presence would indicate successful active loading [42]. After ELV synthesis, post-processing was performed to stabilize the ELVs, deplete larger particles and unencapsulated miRNA (Figure 3A). The miR-126 cargo was encapsulated in ELVs with an average of 34.9 pg RNA per 1.00 × 10^6^ particles, a large increase compared to sEVs (miR-126 not detected), highlighting the scope of this modified TFH method for selective and tailored cargo enrichment (Figure 3B).

### 3.3. miR-126+ ELVs Are Taken up by CECs and Increase CEC Tube Formation

**Figure 4:**
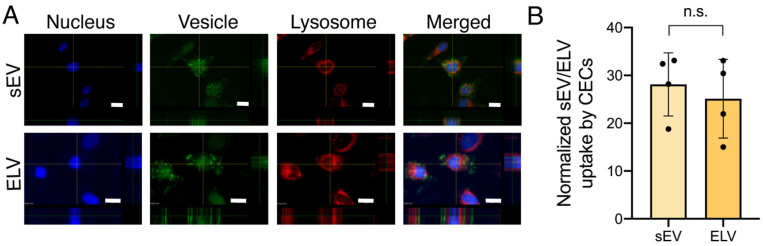
miR-126+ ELVs are successfully taken up by cardiac endothelial cells (CECs). (**A**) Representative images of calcein stained ELV and sEV (green) uptake by 2D CEC cultures labeled for nuclei (blue) and lysosome (red). Images were obtained by confocal laser scanning microscopy from central focal plane with orthogonal images on right and bottom. Scale bar = 10 µm. (**B**) Quantification of uptake of calcein+ ELVs and sEVs by CECs through flow cytometry. Data normalized to negative control. *n* = 4 represent biological replicates. Mean ± SEM. Significance was tested with two-way Student’s paired *t*-test. n.s. = not significant.

**Figure 5:**
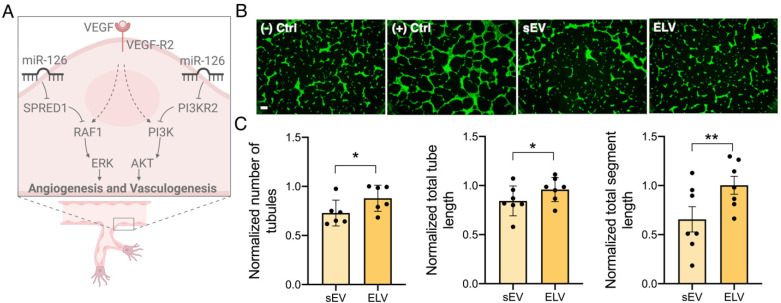
miR-126+ ELVs induce pro-angiogenic response in CECs. (**A**) Schematic of miR-126 mechanism of action for angiogenesis. VEGF: vascular endothelial growth factor; RAF1: rapidly accelerated fibrosarcoma proto-oncogene; ERK: extracellular signal-regulated kinase; AKT: protein kinase B; PI3K: Phosphoinositide 3-Kinase. (**B**) CECs (green) treated with miR-126+ ELVs or sEVs incubated on Matrigel form tubes after overnight incubation. CECs treated with calcein-AM. Scale bar = 200 µm. (**C**) Quantification of angiogenic parameters (ImageJ software version 2.1.0/1.53 g) shows increase in number of tubules, total tube length and segment length for CECs treated with miR-126+ ELVs compared to sEVs. Data normalized to negative control. *n* = 6–7 represent biological replicates. Mean ± SEM. Significance was tested with two-way Student’s paired *t*-test. * *p* ≤ 0.05, ** *p* ≤ 0.01.

miR-126+ ELV uptake by CECs was confirmed qualitatively with confocal laser scanning microscopy (Figure 4A). Calcein+ ELVs are taken up by the CECs, similar to sEVs, without primary trafficking into the lysosome for degradation. Quantitative analysis of ELV and sEV uptake through flow cytometry shows ELVs were taken up to a similar extent to that of sEVs, with no statistical difference between ELV and sEV internalization (Figure 4B). Further, treatment of CECs with ELVs or sEVs did not affect CEC viability, indicating miR-126+ ELVs are not cytotoxic (Supplementary Figure S1). Next, to validate the functional effect of miR-126+ ELVs, the angiogenic potential was assessed through the endothelial tube formation assay. miR-126 is implicated in angiogenesis through the ERK and AKT pathways by targeting SPRED1 and PI3KR2 (Figure 5A). The addition of both sEVs and ELVs induced the formation of tubes by CECs on the Matrigel^®^ Matrix (Figure 5B). However, treatment with ELVs increased the formation of tubules, tube length and segment length compared to sEVs (Figure 5C). This underscores the functional benefit and scope of customized cargo loading into vesicles.

## 4. Discussion

CPC-sEVs are important mediators of cell–cell communication and specifically contain cardioprotective RNA and miRNA cargo crucial for cell-based cardiac repair after MI. Despite their therapeutic benefits, sEV cargo content is often variable, with limited external control of cargo composition. Synthetic mimics such as SUVs allow for such cargo modulation but suffer other challenges such as low stability and rapid clearance when administered, possibly attributed to their simpler membrane composition. In this study, we show the successful synthesis of CPC-sEV derived ELVs with select miRNA using TFH to combine the benefits of the sEV membrane with that of the SUV’s custom cargo loading. Further, the results show the ability of such selective miRNA-loaded ELVs to be internalized by CECs and their functional scope to initiate pro-angiogenic effects in vitro. These findings underscore the potential of ELVs to be potent and tunable alternatives to sEVs for cardiac therapies, and the value of further exploration in this field.

Different methods to exogenously modify sEV cargo were studied, such as sonication, saponification, extrusion, electroporation, and parent-cell transfection. However, these largely focus on slight cargo modifications, suffer inconsistent loading and typically do not clear inherent sEV cargo first, making it difficult to develop reliable and consistent miRNA-loaded sEV cardiac therapeutics. TFH is a well-established process for the synthesis of SUVs and other synthetic vesicle mimics that utilizes the creation of a lipid film. To address some of the limitations of purely artificial SUVs, hybrids of SUVs and sEVs were created utilizing TFH and extrusion with sEVs. Specifically, hybrids designed from CPC-sEVs increased the activity of AKT, a downstream target of miR-126 [43]. In this study, we take this idea of hybrids one step further, by utilizing a completely sEV-based membrane to form our sEV-scale ELVs (Figure 2). With our modified TFH approach we allow for ELV creation from sEVs instead of pure lipids and aim to minimize the presence of inherent sEV cargo, to improve cargo consistency. Given the high encapsulation efficiency of TFH and the ability to modify TFH to develop sEV membrane-derived vesicles, the scope of TFH as a tool for exogenously modified sEVs is present. Thus, TFH can now be utilized to design other exogenously modified vesicles, as a counterpart to currently established methods such as sonication, saponification, etc. Beyond this, the modified TFH method established here also allows for versatility in synthesis by allowing ELV engineering from any parent cell type-derived sEVs beyond CPC-sEVs. However, given the batch-to-batch variability of TFH and the ELV self-assembly process during rehydration in an aqueous buffer, larger starting samples may be required for large-scale ELV production.

An important aspect of sEV-based therapies is sEV uptake or endocytosis for cargo trafficking. Several mechanisms of sEV uptake were established including direct fusion, clathrin or caveolin-mediated endocytosis, lipid-raft mediated endocytosis and macropinocytosis [44]. Although the role of exact membrane components is underexplored, sEV membrane components such as phosphatidylserine were implicated in sEV uptake processes [45]. Further, given the challenges of systemic clearance with synthetic sEV mimics, this further highlights the potential benefit of an sEV-like membrane in ELV composition. Here, we show that the uptake of ELVs is similar to that of sEVs, despite their modified cargo, without active trafficking into the lysosome for degradation (Figure 4A,B) which indicates the importance of the vesicle membrane and warrants further study.

Upon internalization, the cargo composition of sEVs can drastically alter the cardioprotective effects of the sEV therapies [46]. Specifically, several miRNAs were associated with angiogenesis, anti-fibrosis and ischemic recovery after MI, but the incorporated miRNA profile is dependent on external factors such as parent cell type (e.g., CPC or mesenchymal stromal cell), age and culture conditions, limiting our control over the cargo [19,20,47]. Further, despite their benefit, miRNA yield in sEVs is very limited, minimizing sEV potency [21]. For this study, miR-126 was specifically selected as it is pro-angiogenic, involved in endothelial cell survival and repair, anti-inflammatory, anti-fibrotic and anti-hypertrophic, making it a favorable miRNA for myocardial recovery after ischemia [48,49,50,51] The encapsulation of miR-126 in TFH-synthesized ELVs allows incorporation of miRNA of choice, not only enriching pro-reparative miRNA but also loading cardioprotective miRNA with low expression levels in the corresponding sEVs (Figure 3B).

Prior studies of CPC-sEVs have shown their pro-angiogenic potential and several inherent sEV-miRNA associated with angiogenic function [17]. Here, the results show that the functional effect of miR-126+ ELVs was also confirmed through endothelial tube formation (Figure 5B and C). Interestingly, ELVs increased tube formation parameters compared to sEVs despite being actively loaded with only one miRNA. This highlights the further scope of ELV-based therapies, particularly if higher doses or multiple miRNAs are loaded into the ELVs, allowing simultaneous targeting of several MI-relevant outcomes (e.g., fibrosis, inflammation, hypertrophy). Further, with recent advances in RNA sequencing, several miRNAs associated with improvements in myocardial outcomes were established [17,19,20]. This knowledge allows for most targeted and customized miRNA combinations to be loaded into ELV for repair after MI. In addition, ELVs also provide a vehicle for experimentally validating this repository of cardioprotective miRNA.

This study provides the groundwork for combining the benefits of sEV membranes and modulating inherent cargo. TFH allows for tailored miRNA-loaded ELV synthesis with functional improvements compared to sEVs. However, given the need for organic solvents in TFH lipid film creation, there is a possibility that sEV membrane proteins are denatured in ELVs. As membrane proteins can enhance cell-specific targeting of vesicles and immune evasion, attempts to preserve or re-incorporate membrane proteins would be valuable for minimally invasive, cardiac-specific ELVs. Moreover, additional study of the exact roles of different CPC-sEV membrane lipids and proteins and the effect of inner and outer leaflet lipid presence in sEV trafficking and uptake by cardiac cells will allow better selection of sEVs for ELV synthesis. In summary, an improved understanding of sEV membrane components and selection of pro-reparative miRNA for MI treatment will allow further advancement of the TFH-based ELVs engineered in this study.

## 5. Conclusions

The ability to create vesicles with an sEV-like structure and membrane that permits tailored cargo loading provides versatility in the field of vesicle-based cardiac therapeutics. Here, we establish a proof-of-concept design by combining the benefits of sEVs and their synthetic mimics, to modify encapsulated cargo and improve in vitro angiogenic outcomes. Beyond this, ELVs also have the potential to carry different RNA combinations and can be packaged to deliver protective cues for several other diseases, even beyond MI.

## Supplementary Materials

The following are available online at https://www.mdpi.com/article/10.3390/jcdd8110135/s1, Figure S1: Cytotoxicity of miR-126+ ELVs when administered to CECs.
