# Supplementary material for: Engineering Cardiac Small Extracellular Vesicle-Derived Vehicles with Thin-Film Hydration for Customized microRNA Loading
Source: J Cardiovasc Dev Dis. 2021 Oct 22;8(11):135. doi: 10.3390/jcdd8110135 (PMC8626043; doi:10.3390/jcdd8110135)
Supplement: Supplementary file 1 [file jcdd-08-00135-s001.zip › jcdd-1383438-supplementary.pdf]

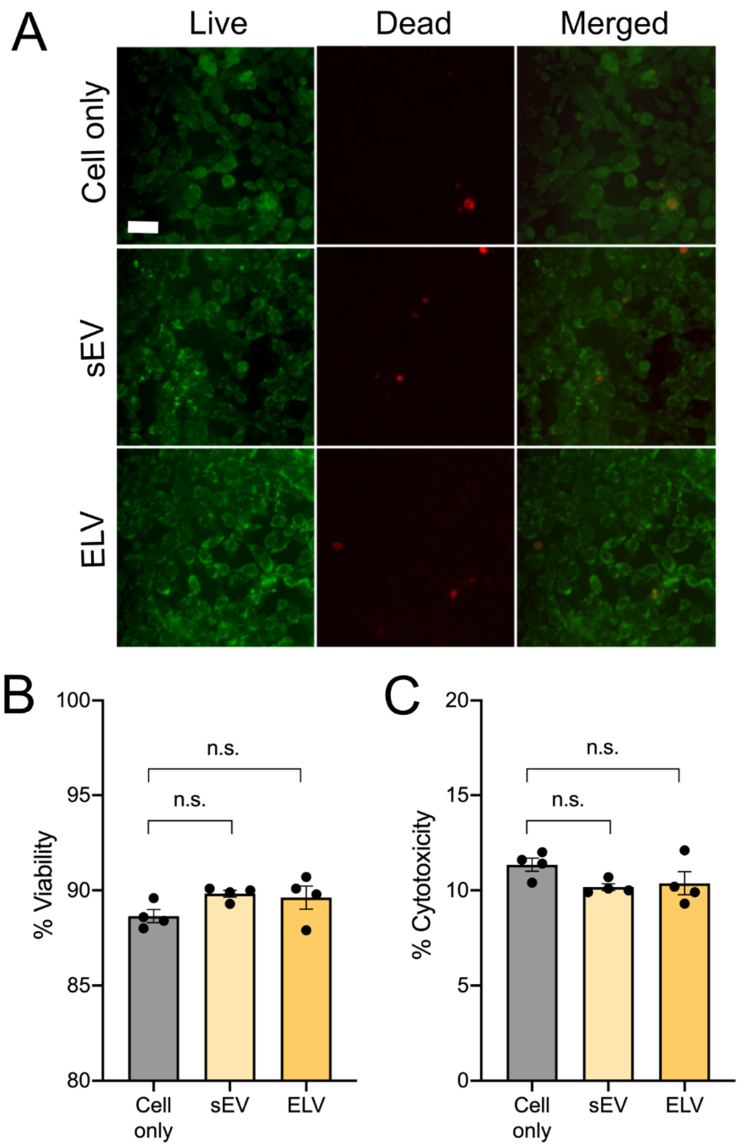

Supplementary Figure S1. Cytotoxicity of miR-126+ ELVs when administered to CECs. (**A**) Cell viability images of CECs after 48 hours incubation with miR-126+ ELVs or sEVs as measured by fluorescence microscopy. Green (live): calcein-AM and red (dead): ethidium homodimer-1. (**B**) Viability and (**C**) Cytotoxicity of CECs from panel (**A**) as measured with the lactate dehydrogenase assay. Data shows no significant difference in viability or cytotoxicity both qualitatively and quantitatively after administration of ELVs or sEVs. Data normalized to negative control (0.1% Triton-X treated CECs). $n = 4$. Mean ± SEM. Significance was tested with one-way ANOVA. Scale bar = 25 μm.
